# Early Fever Is Associated With Clinical Outcomes in Patients With Coronavirus Disease

**DOI:** 10.3389/fpubh.2021.712190

**Published:** 2021-08-26

**Authors:** Feng-ming Ding, Yun Feng, Lei Han, Yan Zhou, Yong Ji, Hui-juan Hao, Yi-shu Xue, Dong-ning Yin, Zeng-chao Xu, Shan Luo, Peng-yu Zhang, Min Zhang

**Affiliations:** ^1^Department of Respiratory and Critical Care Medicine, Shanghai General Hospital, Shanghai Jiao Tong University School of Medicine, Shanghai, China; ^2^Department of Gastroenterology, Shanghai General Hospital, Shanghai Jiao Tong University School of Medicine, Shanghai, China; ^3^School of Mathematical Sciences, Shanghai Jiao Tong University, Shanghai, China; ^4^Department of Infectious Disease, Shanghai General Hospital, Shanghai Jiao Tong University School of Medicine, Shanghai, China

**Keywords:** early fever, clinical outcomes, COVID-19, severity, interleukin-6

## Abstract

Fever is one of the typical symptoms of coronavirus disease (COVID-19). We aimed to investigate the association between early fever (EF) and clinical outcomes in COVID-19 patients. A total of 1,014 COVID-19 patients at the Leishenshan Hospital were enrolled and classified into the EF and non-EF groups based on whether they had fever within 5 days of symptom onset. Risk factors for clinical outcomes in patients with different levels of disease severity were analyzed using multivariable analyses. Time from symptom onset to symptom alleviation, CT image improvement, and discharge were longer for patients with moderate and severe disease in the EF group than in the non-EF group. Multivariable analysis showed that sex, EF, eosinophil number, C-reactive protein, and IL-6 levels were positively correlated with the time from symptom onset to hospital discharge in moderate cases. The EF patients showed no significant differences in the development of acute respiratory distress syndrome, compared with the non-EF patients. The Kaplan–Meier curve showed no obvious differences in survival between the EF and non-EF patients. However, EF patients with increased temperature showed markedly lower survival than the non-EF patients with increased temperature. EF had no significant impact on the survival of critically ill patients, while an increase in temperature was identified as an independent risk factor. EF appears to be a predictor of longer recovery time in moderate/severe COVID-19 infections. However, its value in predicting mortality needs to be considered for critically ill patients with EF showing increasing temperature.

## Introduction

Coronavirus disease (COVID-19) is a serious respiratory disorder that was initially discovered in Wuhan, China. COVID-19 has been shown to be caused by infection with severe acute respiratory syndrome coronavirus (SARS-CoV)-2, a novel coronavirus from the same family as SARS-CoV (1). The CoVs are RNA viruses broadly distributed among mammals and birds; they cause respiratory and intestinal infections in animals and humans (2). Both the severe acute respiratory syndrome (SARS) epidemic in the Guangdong province, China in 2002 and the Middle East respiratory syndrome (MERS) pandemic in the Middle-Eastern countries in 2012 were caused by CoVs (3, 4). SARS-CoV, MERS-CoV, and SARS-CoV-2, which are all β-CoVs, can cause severe respiratory disease in humans (5).

COVID-19 has spread across China and worldwide; the World Health Organization declared it a pandemic within a few months of its first report (6). The patients presented clinical symptoms of dry cough, fever, dyspnea, bilateral lung infiltrates on imaging, and in some cases, gastrointestinal infection symptoms (7). Mild cases typically recover within a week, while patients with more severe forms of the disease show respiratory failure due to alveolar damage and may eventually die (8). There are several factors influencing the outcomes of COVID-19. Non-communicable conditions such as high blood pressure, cardiovascular disease, and diabetes have been reported as risk factors for patients with new coronary disease (9, 10). Patients with hematological malignancies have also been shown to have a higher risk of respiratory infections and serious complications (11).

Fever is an important part of the host defense against infection, playing a key role in increasing the clearance of microorganisms, modulating the cellular immune responses, and inducing heat shock responses (12). During a viral infection, the host mounts an immune response addressed to contain the infection, and fever is regarded as a cornerstone diagnostic sign for screening patients that are potentially infected with COVID-19 (13, 14). Most influenza A strains that infect humans are sensitive to temperature and their replication is inhibited under the body temperature range of 38–41°C. Furthermore, during viral infection, the fever response determines the survival advantage (15). Based on a meta-analysis, Hu et al. reported that fever is the most common symptom in 85.6% of COVID-19 cases during the course of the disease (16). Another study showed that patients with no fever on admission had worse outcomes in the critical/mortality group (17). However, to our knowledge, thus far, no study has focused on the association between early fever (EF) and clinical outcomes in a heterogeneous population of COVID-19 patients with different levels of case severity.

In this study, we compared the clinical outcomes between EF and non-EF patients suffering from moderate, severe, and critical COVID-19 and investigated the value of EF in predicting clinical outcomes in patients with different disease severities.

## Materials and Methods

### Ethical Approval

The ethics committee of Leishenshan Hospital approved this study (Approval No. 2020-LS04). Informed consent was obtained from all patients prior to being enrolled in the study.

### Patients

**Figure 1:**
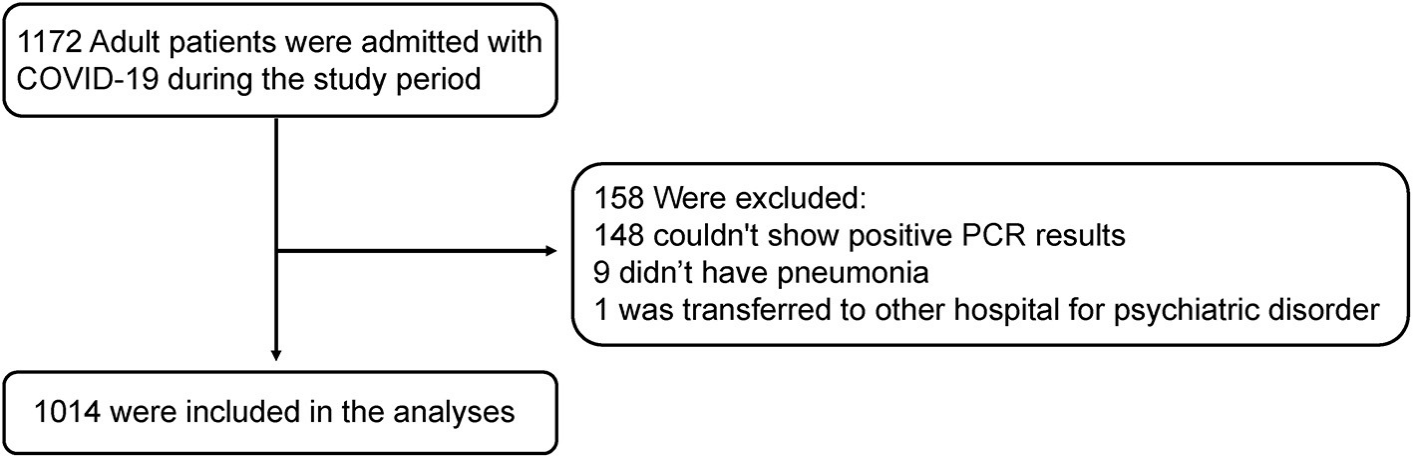
Selection of the study cohort.

A total of 1,172 patients diagnosed with COVID-19 and hospitalized at Leishenshan Hospital, a COVID-19-designated hospital in Wuhan, between February 16 and April 14, 2020, were retrospectively reviewed. All cases were diagnosed and confirmed based on the interim guidance issued by the World Health Organization (18). Only adult patients with positive nucleic acid tests for respiratory specimens (nasopharyngeal or oropharyngeal swab samples) or sputum specimens prior to admission were included. Patients with no pneumonia or patients who were transferred to other hospitals were excluded from our study. Finally, we enrolled 1,014 patients after screening using the pre-determined selection criteria (Figure 1).

Leishenshan Hospital is no longer existing. It was officially closed on April 15, 2020 when the epidemic was got controlled in Wuhan. Indeed, the hospital was a temporary one, which was especially built and used for COVID-19 treatment in Wuhan since the local hospital resources were not enough at that time, and the clinicians were from different hospitals across the country. Some authors of our manuscript (Fengming Ding, Yun Feng, Yan Zhou, Yong Ji, Peng-yu Zhang) were members of Shanghai medical supporting team working in Leishenshan. The medical data in our manuscript were collected and used by our team members. We have no issue of data transferring.

### Study Design

All patients included in the study were classified into EF or non-EF groups based on whether they had fever within 5 days of symptom onset. The symptoms of COVID-19 included cough, dyspnea, sore throat, thoracic tightness or pain, myalgia, headache, diarrhea, nausea, and consciousness disorder. Fever was defined as a maximum body temperature (T_max_) of more than 37.3°C, measured using an ear thermometer. Based on the Clinical Classification in the Novel Coronavirus Pneumonia Diagnosis and Treatment Guidance from the National Health Commission of China (19), the severity of disease was assessed at the first clinic visit. In brief, moderately ill patients were diagnosed based on fever and respiratory symptoms, as well as radiologic manifestations of pneumonia. Severely ill patients were diagnosed using any of these criteria: (1) difficulty breathing, with more than 30 breaths per minute, (2) oxygen saturation levels <93% at rest, and (3) partial arterial oxygen pressure (PaO_2_)/fraction of inspired oxygen (FiO_2_) <300 mmHg (l mmHg = 0.133 kPa). Patients whose chest imaging showed apparent lesion progression of more than 50% within 24–48 h were classified as severe cases. Patients with respiratory failure requiring mechanical ventilation, or shock or organ failure requiring ICU care were classified as critical cases.

### Data Collection

Two researchers independently collected the demographic and clinical information by reviewing electronic medical records, including laboratory data, radiographic characteristics, disease severity, treatment protocols, length of hospital stay, disease progression, and clinical outcomes. Symptom data were also estimated and confirmed by direct communication with patients or their families.

The primary outcome for patients with moderate/severe infection was the time from symptom onset to discharge from hospital. Patients who met the following criteria were discharged from the hospital: significant improvement in clinical symptoms and chest CT imaging, at least two consecutive negative nucleic acid tests from respiratory specimens (nasopharyngeal or oropharyngeal swab samples), and sputum specimens with an interval of more than 24 h. Other clinical outcomes included time from symptom onset to symptom alleviation and the recovery of lymphocyte and eosinophil numbers.

The primary outcome for critically ill patients was the time from symptom onset to death. Other clinical outcomes included time from symptom onset to incidence of acute respiratory distress syndrome (ARDS) and complications (i.e., shock, disseminated intravascular coagulation, and multiple organ failure).

### Statistical Analysis

Data were analyzed using SAS software (version 8.0; SAS Institute, Cary, NC, USA). GraphPad Prism (version 5; GraphPad Software, San Diego, CA, USA) and R software version 4.0.2 (http://CRAN.R-project.org, R Foundation, Vienna, Austria) were used to generate graphs for visualizing the results. The Kolmogorov-Smirnov test was used to determine the distribution of each variable. Normally distributed quantitative variables were compared using independent *t*-test, while non-normal distributed variables were compared using Mann-Whitney-test. Qualitative variables were analyzed using chi-squared test. Differences between end-hospitalization and the first clinic visit for each index are shown using Δindex(a) [index(a) at end-hospitalization subtracting index(a) at the first clinic visit]. ΔT_max_ is the T_max_ in the last 3 days before end-hospitalization minus the T_max_ from symptom onset to the first clinic visit. Data on demographic, clinical and laboratory tests, and treatments were included in a generalized linear model to estimate correlations between EF and time from symptom onset to hospital discharge in patients with moderate/severe infections. The outcomes of ARDS, complications, and survival in critically ill patients were analyzed using the Kaplan-Meier method followed by the log-rank test to calculate differences between the two groups. A Cox proportional-hazards regression model with stepwise selection was used to identify risk factors for mortality in critical patients. Pairwise Spearman correlations (*r*) were calculated between indices at two time points (the first clinic visit and end-hospitalization) in EF and non-EF patients. Results with absolute correlations |*r*| > 0.2 were utilized, and the network was visualized using line widths matched to the strength of correlation. We selected a *p-*value < 0.05 to denote statistically significant differences.

## Results

### Patient Characteristics

Baseline characteristics (at the first clinic visit) of the 1,014 patients are shown in Table 1. Patients with EF accounted for 66.6% (*n* = 675) of the study participants, while non-EF patients made up 33.4% (*n* = 339) of the participants. EF patients had a mean age of 56.6 ± 15.0 years, and 48.7% were female; non-EF patients were aged 58.8 ± 14.3 years, and 56.3% were female. EF patients tended to be more prone to cough symptoms (66.5 vs. 57.9%, χ^2^ = 7.38, *P* = 0.0066), myalgia (24.3 vs. 11.8%, χ^2^ = 21.93, *P* < 0.0001), and diarrhea (9.2 vs. 4.4%, χ^2^ = 7.29, *P* = 0.0069) compared with non-EF patients. A higher proportion of non-EF patients had comorbidities such as chronic lung diseases (6.2 vs. 2.1%, χ^2^ = 11.50, *P* = 0.0007), cardiovascular disease (12.7 vs. 8.4%, χ^2^ = 4.56, *P* = 0.0327), and chronic kidney failure (8.3 vs. 4.4%, χ^2^ = 6.09, *P* = 0.0136) relative to patients with EF. In addition, EF patients had higher levels of C-reactive protein (CRP, *t* = 6.34, *P* < 0.0001), and IL-6 (*t* = 7.51, *P* < 0.0001) at the first clinic visit compared with non-EF patients (*P* < 0.05).

**Table 1:** Demographics and baseline characteristics of COVID-19 patients with or without early fever.

**Characteristic**	**Moderate**				**Severe**				**Critical**			
	**EF**	**Non-EF**	*t -*value^*^	***P*-value**	**EF**	**Non-EF**	*t -*value^*^	***P*-value**	**EF**	**No EF**	*t -*value^*^	***P*-value**
	**(*N* = 440)**	**(*N* = 229)**	or χ^**2**^-value^**^		**(*N* = 181)**	**(*N* = 79)**	or χ^**2**^-value^**^		**(*N* = 54)**	**(*N* = 31)**	or χ^**2**^-value^**^	
Age, years (SD)	54.3 (14.7)	56.3 (14.4)	1.69^*^	0.0908	58.9 (14.3)	62.7 (12.4)	1.98^*^	0.0484	67.6 (13.8)	67.5 (12.8)	0.08^*^	0.9387
<60 years, no. (%)	275 (62.5)	130 (56.8)	2.07^**^	0.1501	87 (48.1)	32 (40.5)	1.27^**^	0.2605	14 (25.9)	7 (22.6)	0.12^**^	0.7307
≥60 years, no. (%)	165 (37.5)	99 (43.2)			94 (51.9)	47 (59.5)			40 (74.1)	24 (77.4)		
Female, no. (%)	212 (48.2)	136 (59.4)	7.58^**^	0.0060	102 (56.4)	42 (53.2)	0.23^**^	0.6342	15 (27.8)	13 (41.9)	1.79^**^	0.1813
Time from symptom onset to the first clinic visit, days, median (IQR)	4 (2–7)	4 (2–7)	0.43^*^	0.5831	5 (2–7)	6 (2–7)	1.03^*^	0.1547	5 (1–7)	4 (1–7)	0.52^*^	0.3971
**Symptoms, no. (%)**												
Cough	292 (66.4)	127 (55.5)	7.65^**^	0.0057	124 (68.5)	50 (63.3)	0.68^**^	0.4109	33 (61.1)	19 (61.3)	0.01^**^	0.9870
Dyspnea	116 (26.4)	50 (21.8)	1.66^**^	0.1981	102 (56.4)	41 (51.9)	0.44^**^	0.5066	23 (42.6)	18 (58.1)	1.89^**^	0.1694
Sore throat	32 (7.3)	24 (10.5)	2.02^**^	0.1552	11 (6.1)	8 (10.1)	1.33^**^	0.2486	1 (1.9)	2 (6.5)	1.22^**^	0.2686
Thoracic tightness or pain	21 (4.8)	14 (6.1)	0.55^**^	0.4599	7 (3.9)	2 (2.5)	0.29^**^	0.5879	6 (11.1)	1 (3.2)	1.62^**^	0.2030
Myalgia	104 (23.6)	27 (11.8)	13.4^**^	0.0002	53 (29.3)	11 (13.9)	6.99^**^	0.0082	7 (13.0)	2 (6.5)	0.88^**^	0.3477
Headache	18 (4.1)	10 (4.4)	0.03^**^	0.8657	5 (2.8)	2 (2.5)	0.01^**^	0.9158	2 (3.7)	1 (3.2)	0.01^**^	0.9085
Diarrhea	46 (10.5)	12 (5.2)	5.17^**^	0.0230	14 (7.7)	2 (2.5)	2.58^**^	0.1084	2 (3.7)	1 (3.2)	0.01^**^	0.9085
Nausea	16 (3.6)	6 (2.6)	0.49^**^	0.4843	9 (5.0)	5 (6.3)	0.20^**^	0.6558	1 (1.9)	2 (6.5)	1.22^**^	0.2686
Consciousness disorder	0 (0.0)	0 (0.0)	/	/	1 (0.6)	0 (0.0)	0.58^**^	0.4460	5 (9.3)	5 (16.1)	0.90^**^	0.3440
**Comorbidities, no. (%)**												
Chronic lung disease^***^	6 (1.4)	8 (3.5)	3.33^**^	0.0678	5 (2.8)	9 (11.4)	6.28^**^	0.0122	3 (5.6)	4 (12.9)	1.41^**^	0.2356
Hypertension	124 (28.2)	63 (27.5)	0.03^**^	0.8544	67 (37.0)	37 (46.8)	2.21^**^	0.1372	30 (55.6)	15 (48.4)	0.41^**^	0.5239
Diabetes	52 (11.8)	21 (9.2)	1.08^**^	0.2973	22 (12.2)	25 (31.6)	14.11^**^	0.0002	15 (27.8)	7 (22.6)	0.28^**^	0.5985
Cardiovascular disease	22 (5.0)	14 (6.1)	0.37^**^	0.5448	23 (12.7)	18 (22.8)	4.21^**^	0.0403	12 (22.2)	11 (35.5)	1.75^**^	0.1853
Chronic kidney failure	12 (2.7)	4 (1.7)	0.62^**^	0.4309	13 (7.2)	17 (21.5)	11.07^**^	0.0009	5 (9.3)	7 (22.6)	2.88^**^	0.0895
Chronic liver disease	12 (2.7)	12 (5.2)	2.75^**^	0.0973	12 (6.6)	6 (7.6)	0.08^**^	0.7780	7 (13.0)	0 (0.0)	4.38^**^	0.0364
Cancer	5 (1.1)	4 (1.7)	0.42^**^	0.5156	2 (1.1)	0 (0.0)	0.88^**^	0.3483	2 (3.7)	2 (6.5)	0.33^**^	0.5647
**Medications, no. (%)**												
Statin	9 (2.0)	9 (3.9)	2.04^**^	0.1529	5 (2.8)	3 (3.8)	0.20^**^	0.6567	5 (9.3)	2 (6.5)	0.21^**^	0.6504
ACE inhibitor or ARB	35 (8.0)	17 (7.4)	0.06^**^	0.8077	10 (5.5)	6 (7.6)	0.41^**^	0.5229	5 (9.3)	2 (6.5)	0.21^**^	0.6504
Anticoagulant	13 (3.0)	6 (2.6)	0.06^**^	0.8048	9 (5.0)	3 (3.8)	0.17^**^	0.6779	4 (7.4)	3 (9.7)	0.13^**^	0.7140
Hypoglycemic agent	32 (7.3)	13 (5.7)	0.61^**^	0.4343	12 (6.6)	20 (25.3)	17.79^**^	<0.0001	12 (22.2)	4 (12.9)	1.12^**^	0.2901
Systemic steroid	13 (3.0)	2 (0.9)	2.98^**^	0.0845	11 (6.1)	3 (3.8)	0.56^**^	0.4538	7 (13.0)	3 (9.7)	0.20^**^	0.6509
**Vital signs, median (IQR)**												
Body temperature by ear thermometer, °C	38.0 (37.9–38.7)	36.5 (36.2–36.8)	9.44^*^	<0.0001	38.3 (38.0–38.9)	37.0 (36.8–37.0)	20.42^*^	<0.0001	38.0 (38.0–39.0)	37.0 (36.7–37.0)	12.12^*^	<0.0001
Systolic blood pressure, mm Hg	130 (121–141)	130 (120–138)	0.67^*^	0.5010	130 (121–141)	133 (121–145)	1.22^*^	0.2203	131 (120–144)	130 (121–145)	0.57^*^	0.5709
Diastolic blood pressure, mmHg	82 (76–91)	80 (75–90)	0.99^*^	0.3182	80 (73–87)	80 (72–90)	0.34^*^	0.7344	80 (71–84)	78 (71–90)	1.67^*^	0.0979
Heart rate, beats/min	88 (80–98)	86 (76–96)	1.64^*^	0.1022	83 (77–98)	86 (76–95)	0.59^*^	0.5571	97 (84–107)	90 (79–103)	1.52^*^	0.1324
Oxygen saturation %	98 (97–98)	98 (97–98)	0.99^*^	0.3219	95 (90–98)	95 (91–98)	0.25^*^	0.8019	92 (86–96)	93 (90–96)	1.70^*^	0.0925
Respiratory rate, breaths/min	20 (18–22)	20 (18–22)	0.68^*^	0.4970	21 (19–23)	21 (18–23)	1.87^*^	0.0630	23 (20–26)	22 (20–25)	0.84^*^	0.4010
**Laboratory tests, median (IQR)**												
Neutrophil, × 10^9^/L	3.1 (2.4–4.0)	3.4 (2.6–4.2)	0.11^*^	0.9157	3.2 (2.5–4.3)	3.7 (2.5–4.7)	0.66^*^	0.5110	6.1 (3.8–9.8)	6.7 (5.4–9.1)	0.39^*^	0.6990
Lymphocyte, × 10^9^/L	1.6 (1.3–2.0)	1.7 (1.4–2.1)	0.26^*^	0.7932	1.5 (1.0–1.9)	1.4 (0.9–1.9)	0.29^*^	0.7745	0.8 (0.6–1.2)	0.8 (0.4–0.9)	1.02^*^	0.3115
Eosinophil, × 10^9^/L	0.12 (0.07–0.18)	0.12 (0.07–0.21)	1.17^*^	0.2420	0.10 (0.05–0.16)	0.09 (0.06–0.17)	0.06^*^	0.9523	0.01 (0.00–0.06)	0.04 (0.01–0.13)	0.78^*^	0.4388
d-Dimer, μg/ml	0.32 (0.19–0.70)	0.28 (0.17–0.53)	0.89^*^	0.3743	0.62 (0.23–1.28)	0.90 (0.37–3.18)	3.31^*^	0.0011	2.92 (0.7–9.2)	3.08 (1.34–6.42)	0.94^*^	0.3523
Lactate dehydrogenase, U/L	178 (158–207)	173 (154–200)	1.91^*^	0.0566	196 (170–228)	201 (168–248)	0.49^*^	0.6249	360 (251–479)	279 (218–392)	1.50^*^	0.1373
Creatinine, μmol/L	64.6 (54.4–74.7)	62.7 (54.9–72.0)	0.72^*^	0.4745	65.1 (53.5–80.3)	65.9 (55.4–102.2)	2.38^*^	0.0180	75.4 (53.2–103.4)	74.7 (53.7–253.1)	2.73^*^	0.0077
Alanine aminotransferase, U/L	25 (16–40)	27 (14–32)	2.92^*^	0.0036	25 (14–36)	20 (12–31)	0.35^*^	0.7230	25 (19–37)	25 (15–45)	1.04^*^	0.3018
Aspartate aminotransferase, U/L	20 (16–27)	23 (15–25)	0.89^*^	0.3761	20 (15–26)	19 (15–25)	0.76^*^	0.4475	33 (22–54)	24 (18–40)	1.07^*^	0.2891
Albumin, g/L	38.2 (35.6–40.3)	38.6 (36.5–40.9)	1.07^*^	0.2837	36.3 (33.6–39.1)	36.2 (32.1–38.8)	0.92^*^	0.3585	30.5 (27.4–33.3)	31.4 (28.4–34.7)	0.62^*^	0.5338
cTnI, ng/L	0.01 (0.01–0.01)	0.01 (0.01–0.01)	1.32^*^	0.1887	0.01 (0.01–0.01)	0.01 (0.01–0.02)	1.06^*^	0.2880	0.03 (0.01–0.08)	0.03 (0.01–0.10)	0.99^*^	0.3234
IL-6, pg/mL	7.95 (2.05–11.50)	2.0 (1.50–2.56)	8.11^*^	<0.0001	13.74 (5.48–19.86)	5.13 (3.31–6.88)	2.03^*^	0.0476	146.8 (21.9–348.1)	134.8 (9.8–182.4)	1.37^*^	0.1728
CRP, mg/L	13.60 (9.61–24.51)	2.50 (0.65–9.20)	9.81^*^	<0.0001	11.40 (3.60–34.26)	5.99 (1.50–24.61)	1.84^*^	0.0669	46.91 (15.12–83.26)	15.18 (4.87–58.40)	1.51^*^	0.1348
PCT, ng/mL	0.03 (0.02–0.05)	0.03 (0.02–0.04)	0.71^*^	0.4805	0.04 (0.03–0.07)	0.05 (0.03–0.23)	1.00^*^	0.3167	0.23 (0.10–0.87)	0.15 (0.08–0.62)	0.56^*^	0.5800
Bilateral involvement of chest radiographs, no. (%)	411 (93.4)	201 (87.8)	6.14^**^	0.0132	174 (96.1)	73 (92.4)	1.61^**^	0.2047	53 (98.1)	31 (100.0)	0.58^**^	0.4460

*COVID-19, coronavirus disease; EF, early fever; PCT, procalcitonin; CRP, high-sensitivity C-reactive protein; IL-6, interleukin-6*.

* *Quantitative variables of normal distribution were compared using independent t-test*.

** *Qualitative variables were analyzed using the Chi-squared test*.

*** *Chronic lung disease was defined as chronic obstructive pulmonary disease, asthma, or chronic bronchitis*.

Based on the clinical classification in the Seventh Version of the Novel Coronavirus Pneumonia Diagnosis and Treatment Guidance from the National Health Commission of China, the 1,014 patients included 669 moderate cases, 260 severe cases, and 85 critical cases. Table 1 shows that the mean age of critical cases was significantly higher than that of severe (*t* = 2.99, *P* = 0.0030) or moderate cases (*t* = 2.68, *P* = 0.0075). That is, disease severity increased with the age of the patient. EF patients in moderate and severe condition had higher IL-6 level than their non-EF counterparts at the first clinic visit (*t* = 8.11, *P* < 0.0001; *t* = 2.03, *P* = 0.0476; respectively), but no significant difference of IL-6 was found between EF and non-EF patients in critical condition (*t* = 1.37, *P* = 0.1728). The CRP level also showed significant difference between EF and non-EF patients in moderate condition (*t* = 9.81, *P* < 0.0001), but no significant difference was found between the two groups in severe and critical condition (*t* = 1.84, *P* = 0.0669; *t* = 1.51, *P* = 0.1348). By the end of the study, 974 patients had been discharged from the hospital, while 40 had died (one severely ill patient and 39 critically ill patients).

### Changes in Body Temperature Following Hospitalization

After admission to the hospital, antiviral therapy was administered to 405 (60.0%) EF patients and 188 (55.5%) non-EF patients (χ^2^ = 2.05, *P* = 0.1526). Antibiotic therapy was administered to 298 (44.1%) EF patients and 144 (42.5%) non-EF patients (χ^2^ = 0.26, *P* = 0.6129). Systemic glucocorticoid treatment was administered to 45 (6.7%) EF patients and 13 (3.8%) non-EF patients (χ^2^ = 3.36, *P* = 0.0670). Among patients who received systemic glucocorticoids, 21 (46.7%) EF patients and 8 (61.5%) non-EF patients were in critical condition (χ^2^ = 0.89, *P* = 0.3449) and of these, 11 (52.4%) EF patients and 4 (50%) non-EF patients presented with increased body temperatures at end-hospitalization.

**Figure 2:**
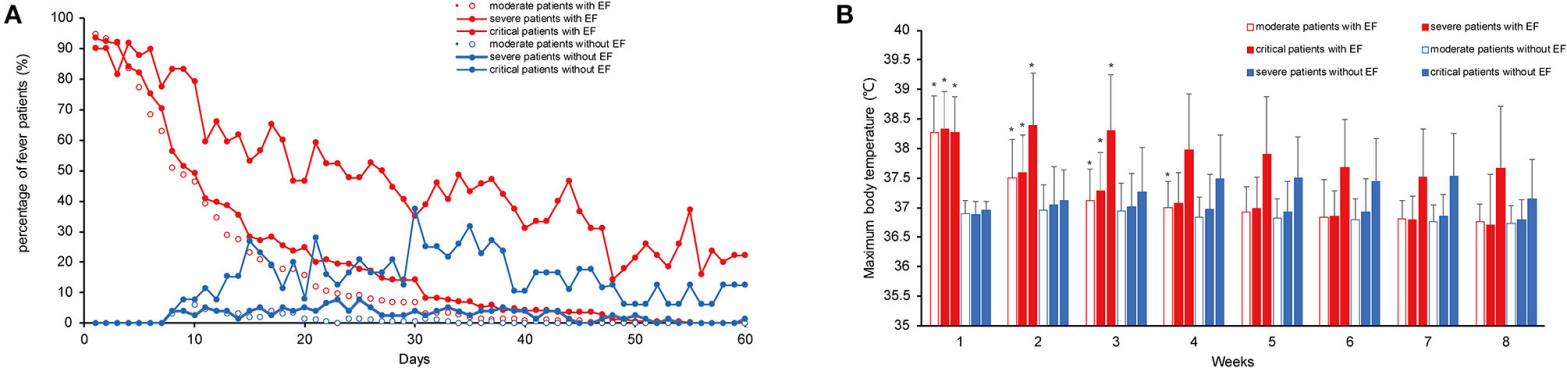
Characteristics of fever in moderate, severe, and critically ill COVID-19 patients with and without EF. Daily percentage of patients with fever in each group **(A)**. Maximum weekly body temperature **(B)**. COVID-19, coronavirus disease; EF, early fever, defined as the incidence of fever (T_max_ ≥ 37.3°C, measured using an ear thermometer) within 5 days of symptom onset. Data are presented as the means ± standard deviations in **(B)**. *P < 0.05 for the EF group vs. the non-EF group in the corresponding severity conditions.

The number of patients with fever in the EF group was significantly reduced after treatment. The number of patients with fever among the severe and moderate cases declined faster than among the critical cases. In the non-EF group, patients developed fever during hospitalization, and most of these were critical cases (Figure 2A). There were no obvious differences in T_max_ between EF and non-EF patients after 4 weeks of hospitalization (Figure 2B).

### Clinical Outcomes in Severe and Moderate Cases

**Figure 3:**
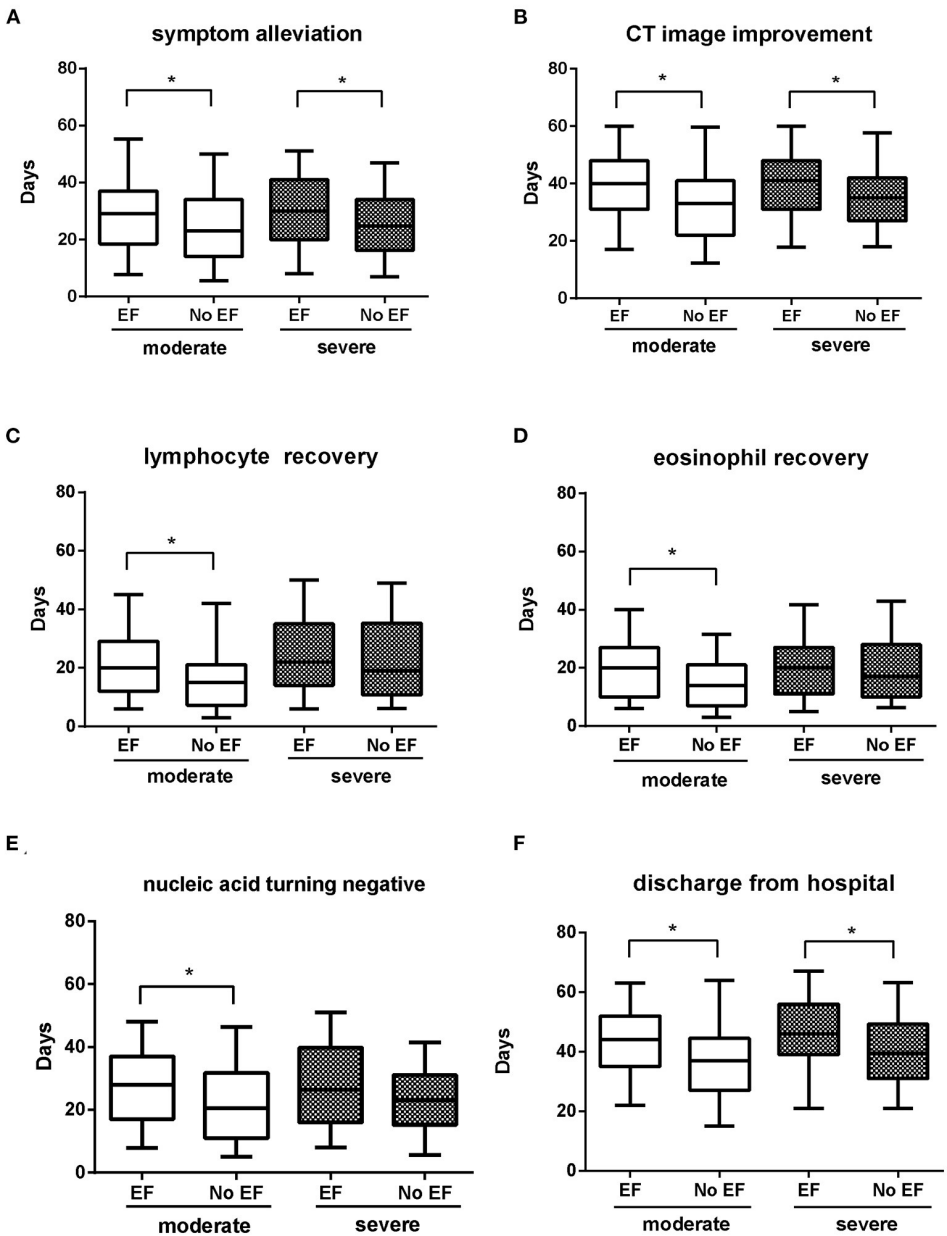
Comparison of clinical outcomes in EF and non-EF COVID-19 patients with moderate/severe infection. Time from symptom onset to symptom alleviation **(A)**; CT image improvement **(B)**; lymphocyte recovery **(C)**; eosinophil recovery **(D)**; nucleic acid tests turning negative **(E)**; time of discharge from hospital **(F)**. Whiskers of the boxplot mark the 5th and 95th percentiles, while the box contains 25th percentile, median, and 75th percentiles. COVID-19, coronavirus disease; EF, early fever, defined as the incidence of fever (T_max_ ≥ 37.3°C, measured using an ear thermometer) within 5 days of symptom onset. *P < 0.05 for EF patients vs. non-EF patients in corresponding severity conditions.

Among the moderately ill cases, patients with EF showed longer time from symptom onset to symptom alleviation, CT image improvement, lymphocyte and eosinophil recovery, nucleic acid tests turning negative, and discharge from hospital compared with non-EF patients (all *P* < 0.05). Among the severe cases, patients with EF showed longer time periods from symptom onset to symptom alleviation, CT image improvement, and discharge from hospital compared with non-EF patients (all *P* < 0.05). No obvious differences were detected in the time from symptom onset to nucleic acid tests turning negative, or to lymphocyte or eosinophil recovery (Figure 3).

### Risk Factors for Time From Symptom Onset to Discharge in Moderately or Severely Ill Patients

The primary outcome for moderately or severely ill patients was the time from symptom onset to discharge from hospital. Therefore, we investigated the factors influencing time from symptom onset to hospital discharge in these cases. Demographic, clinical and laboratory tests, and treatments were included in the generalized linear model (Table 2). Multivariable analysis showed that sex, EF, eosinophil number, CRP, and IL-6 levels were correlated with time from symptom onset to hospital discharge among the moderate cases. CRP levels at first clinic visit showed the highest positive correlation with time from symptom onset to hospital discharge (*t* = 4.26, *P* < 0.0001), followed by EF (*t* = 3.01, *P* = 0.0028) and IL-6 (*t* = 3.00, *P* = 0.0028). Among the severe cases, EF showed the most significant correlation with time from symptom onset to hospital discharge (*t* = 2.42, *P* = 0.0164), followed by IL-6 (*t* = 2.07, *P* = 0.0398). When we compared the results of multivariable analysis between moderately/severely ill patients, the difference in EF coefficient did not reach statistical significance (*t* = 1.12, *P* = 0.2647).

**Table 2:** Multivariable analysis of time from symptom onset to hospital discharge in COVID-19 patients with moderate and severe infections.

**variables**	**Moderate patients**			**Severe patients**		
	**Coefficient (SE)**	***t***	***P-*value**	**Coefficient (SE)**	***t***	***P*-value**
Gender (male = 1, female = 0)	−1.16 (0.54)	−2.13	0.0335	−0.02 (0.92)	−0.02	0.9870
EF (yes = 1, no = 0)	1.83 (0.61)	3.01	0.0028	7.83 (3.23)	2.42	0.0164
Neutrophil (10^9^/L)	0.50 (0.52)	0.96	0.3391	−0.00 (0.02)	−0.09	0.9309
Lymphocyte (10^9^/L)	−0.49 (0.54)	−0.91	0.3610	−0.09 (0.33)	−0.27	0.7859
Eosinophil (10^9^/L)	1.26 (0.53)	2.4	0.0167	−0.19 (0.21)	−0.9	0.3691
CRP (mg/L)	2.47 (0.58)	4.26	<0.0001	−0.04 (0.02)	−1.69	0.0923
PCT (ng/mL)	0.86 (0.51)	1.69	0.0919	−0.06 (0.55)	−0.11	0.9140
IL-6 (pg/mL)	1.78 (0.59)	3.00	0.0028	0.12 (0.06)	2.07	0.0398

*COVID-19, coronavirus disease; EF, early fever; PCT, procalcitonin; CRP, high-sensitivity C-reactive protein; IL-6, interleukin-6; SE, standard error. A generalized linear regression model was developed, and the p-values for the coefficients indicate whether these relationships are statistically significant*.

### Clinical Outcomes in Critical Cases

**Figure 4:**
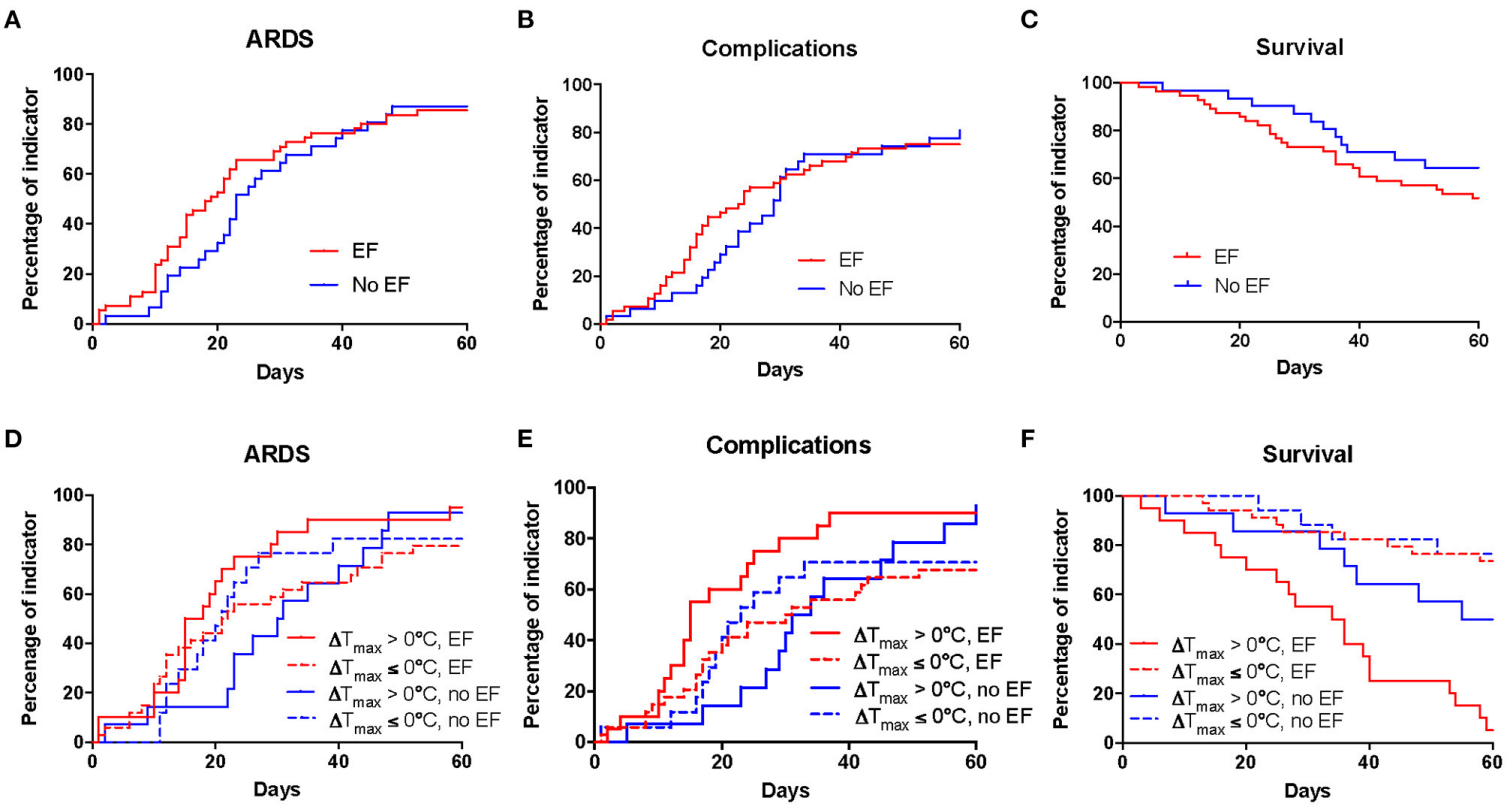
Kaplan-Meier analysis of clinical outcomes in critical COVID-19 patients. Kaplan–Meier curves between EF and non-EF patients for ARDS **(A)**, complications **(B)**, and survival **(C)** were analyzed. The Kaplan-Meier curves of EF and non-EF patients for ARDS **(D)**, complications **(E)**, and survival **(F)** after further dividing patients based on ΔT_max_. T_max_, maximum body temperature; ΔT_max_, the difference between T_max_ in the last 3 days before end-hospitalization and T_max_ from symptom onset to the first clinic visit; COVID-19, coronavirus disease; EF, early fever, defined as the incidence of fever (T_max_ ≥ 37.3°C, measured using an ear thermometer) within 5 days of symptom onset. Complications included shock, disseminated intravascular coagulation, and multiple organ failure.

Clinical outcomes for critically ill patients included time from symptom onset to death, time from symptom onset to incidence of ARDS, and complications. ARDS developed in 74 patients, including 47 (87%) EF patients and 27 (87.1%) non-EF patients. Three patients with acute exacerbation died without ventilator treatment. Among these, 17 EF patients and 12 non-EF patients received non-invasive respiratory support, including high-flow nasal cannula oxygen therapy (4 EF patients and 8 non-EF patients) and non-invasive mechanical ventilation (13 EF patients and 4 non-EF patients). In addition, 29 EF patients and 13 non-EF patients had invasive respiratory support, including intubation and invasive mechanical ventilation (23 EF patients and 12 non-EF patients) and membrane oxygenation (6 EF patients and 1 non-EF patient). EF patients showed no significant differences in the development of ARDS compared with non-EF patients (HR = 1.13, 95% CI 0.71–1.81, *P* = 0.6031, Figure 4A).

A total of 63 patients developed complications, including 40 (74.1%) EF patients and 23 (74.2%) non-EF patients, with no significant differences in the development of complications between EF and non-EF patients (HR = 1.14, 95% CI 0.70–1.86, *P* = 0.6002, Figure 4B). Complications included shock (24 EF patients and 8 non-EF patients), disseminated intravascular coagulation (10 EF patients and 10 non-EF patients), and multiple organ failure (33 EF patients and 23 non-EF patients).

Thirty-nine of the 85 critically ill patients died during the observation period. EF patients accounted for 51.9% (*n* = 28) of these, while 35.5% (*n* = 11) were non-EF patients. The KM curve showed no obvious differences in survival between EF and non-EF patients (HR 1.7, 95% CI 0.85–3.1, *P* = 0.1480, Figure 4C).

EF and non-EF patients were further categorized into the ΔT_max_ > 0°C and ΔT_max_ ≤ 0°C groups. The results showed that EF patients with temperature increase (EF patients with ΔT_max_ > 0°C) had significantly reduced survival compared with non-EF patients with ΔT_max_ > 0°C (HR 3.10, 95% CI 1.42–6.64, *P* = 0.0055), while no obvious difference in survival was detected between EF patients with ΔT_max_ ≤ 0°C and non-EF patients with ΔT_max_ ≤ 0°C (HR 1.17, 95% CI 0.37–3.66, *P* = 0.7956). This suggests that an increase in temperature was a risk factor for mortality in patients with EF. There were no statistically significant differences in complications (HR 1.89, 95% CI 0.99–4.03, *P* = 0.0606) or ARDS (HR 1.77, 95% CI 0.94–3.79, *P* = 0.0895) between EF patients with temperature increase and non-EF patients with temperature increase (Figures 4D–F).

### Risk Factors for Mortality in Critically Ill Patients

**Figure 5:**
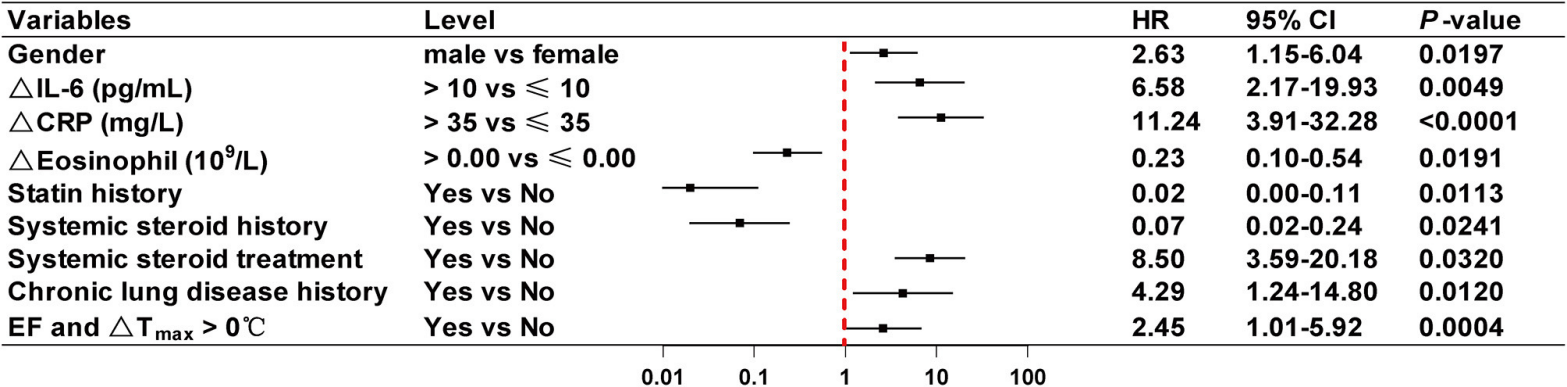
Risk factors for fatal COVID-19 outcome in the multivariate Cox proportional-hazards regression model. Shown in the figure are the hazard ratio (HR) and 95% confidence interval (95% CI) associated with the endpoint. Δ index(a), index(a) end-hospitalization - index(a) at the first clinic visit; T_max_, maximum body temperature; ΔT_max_, the difference between T_max_ in the last 3 days before end-hospitalization and T_max_ from symptom onset to the first clinic visit; COVID-19, coronavirus disease; EF, early fever, defined as the incidence of fever (T_max_ ≥ 37.3°C, measured using an ear thermometer) within 5 days of symptom onset. Chronic lung disease was defined as chronic obstructive pulmonary disease, asthma, or chronic bronchitis.

Using the Cox proportional-hazards regression model with stepwise selection, risk factors for mortality were identified in critically ill patients. Results showed that EF had no significant impact on the survival of critically ill patients, while an increase in temperature (ΔT_max_ > 0°C) was identified as an independent risk factor (HR 4.51, 95% CI 1.31–15.54, *P* = 0.016). Therefore, EF combined with ΔT_max_ > 0°C was included in the regression model. As shown in Figure 5, EF and ΔT_max_ > 0°C had a HR of 2.45 (95% CI 1.01–5.92, *P* = 0.0004), indicating that EF patients with ΔT_max_ > 0°C were at greater risk of mortality than others. In addition, sex (HR 2.63, 95% CI 1.15–6.04, *P* = 0.0197), ΔIL-6 > 10 pg/mL (HR 6.58, 95% CI 2.17–19.93, *P* = 0.0049), ΔCRP > 35 mg/L (HR 11.24, 95% CI 3.91–32.28, *P* < 0.0001), systemic steroid treatment (HR 8.50, 95% CI 3.59–20.18, *P* = 0.0320), and a history of chronic lung disease (HR 4.29, 95% CI 1.24–14.80, *P* = 0.0004) were risk factors for mortality among critical cases. Of these risk factors, ΔCRP > 35 mg/L had the highest hazard ratio.

### Correlation Network Analysis

**Figure 6:**
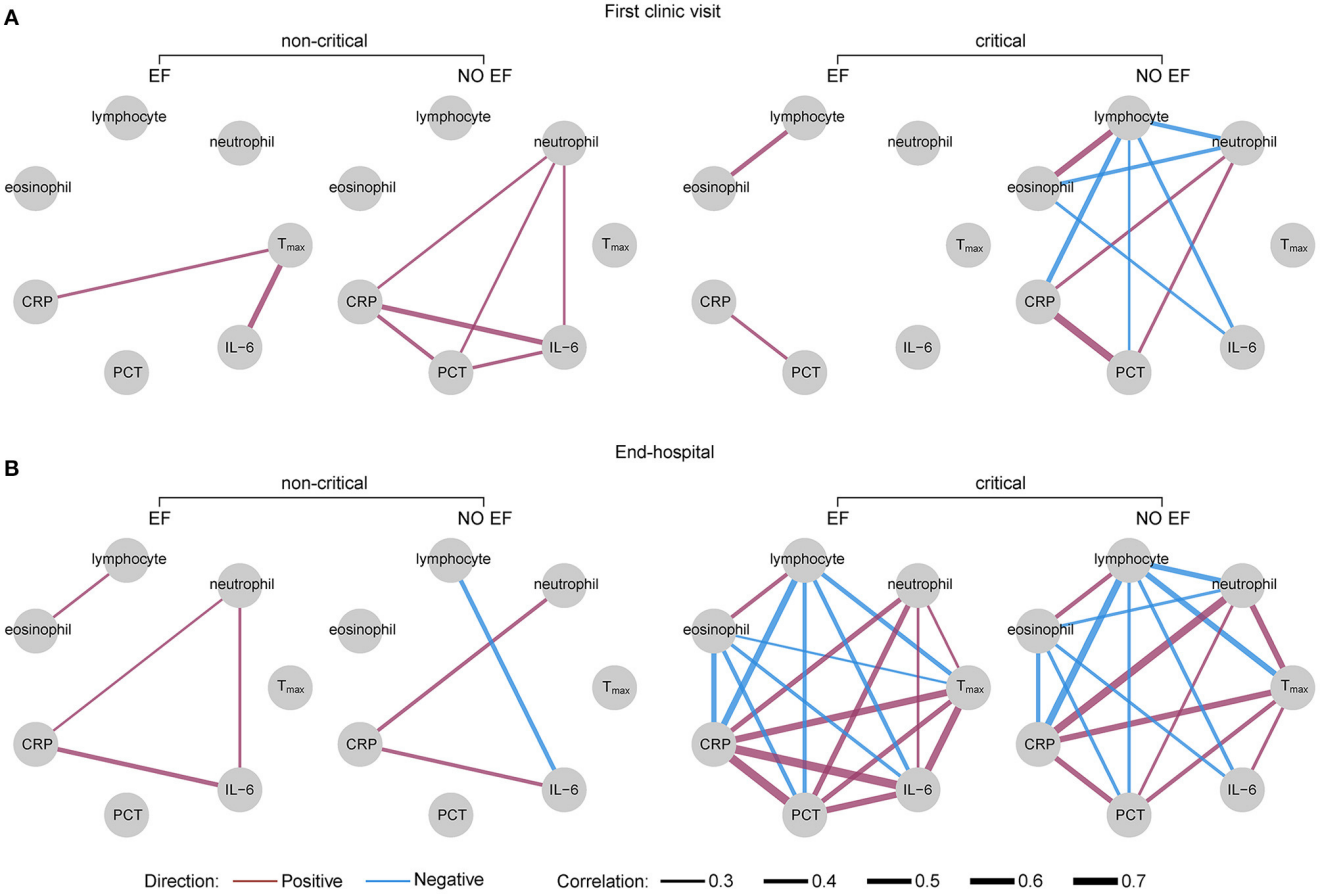
Correlation networks of T_max_, immunologic cells, and biomarkers in EF and non-EF COVID-19 patients. Networks showed different profiles of correlations between EF and non-EF patients in critical and non-critical conditions at the first clinic visit **(A)** and end-hospitalization **(B)**. The width of the edge showing stronger or weaker interactions is proportional to the absolute value of correlation (|*r*|). Edges were observed only when |*r*| > 0.2. A purple edge indicates a positive correlation, and a blue edge indicates a negative correlation. COVID-19, coronavirus disease; EF, early fever, defined as the incidence of fever (T_max_ ≥ 37.3°C, measured using an ear thermometer) within 5 days of symptom onset. T_max_, maximum body temperature from symptom onset to the first clinic visit **(A)**, and T_max_ in the last 3 days before end-hospitalization **(B)**. PCT, procalcitonin; CRP, high-sensitivity C-reactive protein; IL-6, interleukin-6.

The correlation networks between T_max_, the number of immunologic cells, and level of inflammatory biomarkers in EF and non-EF patients are shown in Figure 6. Among moderately/severely ill patients (non-critical), T_max_ showed a moderate correlation with IL-6 level (*r* = 0.48) and a weak correlation with CRP level (*r* = 0.26) in patients with EF at first clinic visit. No correlations were found between T_max_ and both IL-6 and CRP levels in non-EF patients. In addition, there were correlations between CRP, IL-6, and procalcitonin (PCT) levels and neutrophil counts in non-EF patients. At end-hospitalization, T_max_ showed no correlation with immunologic cell numbers or inflammatory biomarker levels in either EF or non-EF patients.

However, the reverse effects were observed in critically ill patients; at the first clinic visit, T_max_ showed no correlation with immunologic cell numbers or inflammatory biomarker levels in both EF and non-EF patients. At end-hospitalization, T_max_ was positively correlated with neutrophil numbers and CRP, IL-6, and PCT levels, but negatively correlated with lymphocyte numbers in both EF and non-EF patients. The correlation between T_max_ and IL-6 levels in EF patients was significantly weaker than in non-EF patients (0.65 vs. 0.27, *z* = −2.12, *P* = 0.0170). In addition, T_max_ was negatively correlated with eosinophil counts (*r* = −0.24) in patients with EF, while no correlation was found between T_max_ and eosinophils in non-EF patients.

## Discussion

Fever is one of the earliest symptoms that COVID-19 patients display when visiting the clinic for treatment, and it is easily measured in these patients (20, 21). In this investigation of clinical outcome in a large sample of COVID-19 patients, we found that EF patients with moderate/severe infection exhibited a longer time period between symptom onset and symptom alleviation, CT image improvement, and discharge from hospital compared with non-EF patients. We also found that EF was significantly positively correlated with time from symptom onset to hospital discharge in patients with moderate/severe infection. In addition, critically ill EF patients whose temperature increased as the disease progressed had worse survival outcomes compared with critically ill, non-EF patients. EF combined with ΔT_max_ > 0°C was an independent risk factor for mortality in critically ill patients.

Considering that mortality rates were quite different between critically and non-critically ill patients (22), the time to hospital discharge or death were selected as the primary outcomes for moderately/severely ill patients and critically ill patients, respectively. Our data showed that 39 (45.9%) of the 85 critically ill patients died during the observation period, while only one severely ill patient died during the observation period. Factors other than EF that were significantly associated with discharge time in patients with moderate/severe disease included sex, levels of IL-6 and CRP, and eosinophil numbers. All these factors, apart from EF, have been reported in previous studies (23–25). Apart from EF combined with temperature increase, risk factors for mortality in critically ill patients included sex (HR 2.63, 95% CI 1.15–6.04, *P* = 0.0197), ΔIL-6 > 10 pg/mL (HR 6.58, 95% CI 2.17–19.93, *P* = 0.0049), ΔCRP > 35 mg/L (HR 11.24, 95% CI 3.91–32.28, *P* < 0.0001), systemic steroid treatment (HR 8.50, 95% CI 3.59–20.18, *P* = 0.0320), and a history of chronic lung disease (HR 4.29, 95% CI 1.24–14.80, *P* = 0.0004). Most of these factors have also been reported in previous studies (26–29). However, to our knowledge, the positive correlations between increased temperature and mortality risk in patients with EF have not been reported.

SARS-CoV-2 infection can stimulate leukocytes to release cytokines, and in critically ill patients, an aberrant uncontrolled response known as “cytokine storm” can be triggered, contributing to lymphopenia, lung injury, and multi-organ failure (30, 31). Of these cytokines, IL-6 is a major pyrogenic cytokine that elevates the core body temperature *via* thermoregulatory autonomic mechanisms and correlates directly with disease severity (30, 32–34). In this study, we found that EF patients with moderate/severe infection displayed higher IL-6 levels than their non-EF counterparts at first clinic visit, and that there were positive associations between T_max_ and IL-6 levels in patients with EF. However, no obvious differences in IL-6 levels were observed at first clinic visit between EF and non-EF patients in critical condition and no correlations between T_max_ and IL-6 levels were found in either EF or non-EF patients at the first clinic visit. This phenomenon could be associated with the high proportion of elderly patients who were identified as being in critical condition in this study. It has been reported that elderly patients with COVID-19 have much higher mortality than younger patients and elderly patients often remain sedated or are slow to respond to infection (35–37). Therefore, the temperatures of critically ill patients may not reflect the acute inflammatory responses in early-stage disease, which is consistent with our finding that EF was not an independent risk factor for mortality in critically ill patients. However, our data showed that at end-hospitalization, T_max_ of critically ill patients in both EF and non-EF groups were positively correlated with IL-6 levels, and the correlation was significantly stronger in EF patients than in non-EF patients. This suggests that at the late stage, higher temperatures in critically ill, EF patients could reflect higher IL-6 levels, contributing to formation of cytokine storm and increasing the mortality risk.

The use of systemic glucocorticoids in COVID-19 patients remains debatable (38). Based on a meta-analysis, Lu et al. reported that glucocorticoids can reduce the duration of fever in COVID-19 patients; however, no benefits were found in terms of mortality (39). In our study, systemic glucocorticoids were administered to ~30% of critically ill patients, and no significant differences in glucocorticoid treatment outcomes were observed between EF and non-EF patients. Although the use of systemic glucocorticoids may decrease body temperature in patients with fever (40), our data showed that about half of critically ill patients treated with systemic glucocorticoid still presented with elevated temperature at end-hospitalization. In the multivariable analysis, both systemic glucocorticoid treatment and the combination of EF and increase in temperature were independent risk factors for mortality, suggesting that the impact of glucocorticoids on body temperature did not interfere with the predictive value of EF in critically ill patients when their body temperature increased.

Our study had some limitations. First, this was a single-center study; thus, our results should be confirmed using data from multi-center or prospective studies. In addition, the application of NSAIDs and physical cooling in patients with fever may have affected T_max_ in the correlation network analysis. However, the impact on the results of the correlation analysis was limited because the NSAIDs and physical cooling were only applied when patients had high fever (>39°C) during hospitalization.

## Conclusions

In conclusion, our findings suggest that EF may be a predictor for a long recovery time in moderately/severely ill patients. In critically ill patients, its value in predicting death needs to be considered in cases of elevated temperature. The positive correlation between T_max_ and IL-6 levels may play a role in the capacity for EF to predict clinical outcomes.

## Data Availability Statement

The raw data supporting the conclusions of this article will be made available by the authors, without undue reservation.

## Ethics Statement

The studies involving human participants were reviewed and approved by the Ethics Committee of Leishenshan Hospital approved this study (Approval No. 2020-LS04). The patients/participants provided their written informed consent to participate in this study.

## Author Contributions

MZ, P-yZ, F-mD, YF, and LH: conceptualization. F-mD, YF, LH, YZ, YJ, H-jH, Y-sX, D-nY, Z-cX, SL, P-yZ, and MZ: data curation. YZ, YJ, H-jH, Y-sX, D-nY, Z-cX, and SL: formal analysis. MZ: funding acquisition. F-mD, YF, and LH: writing-original draft. MZ and P-yZ: writing-review and editing. All authors contributed to the article and approved the submitted version.

## Conflict of Interest

The authors declare that the research was conducted in the absence of any commercial or financial relationships that could be construed as a potential conflict of interest. The reviewer ZX declared a shared affiliation with the authors to the handling editor at the time of the review.

## Publisher's Note

All claims expressed in this article are solely those of the authors and do not necessarily represent those of their affiliated organizations, or those of the publisher, the editors and the reviewers. Any product that may be evaluated in this article, or claim that may be made by its manufacturer, is not guaranteed or endorsed by the publisher.
